# Retention of pediatric patients in care: a study of the Kibera Community Health Center HIV/AIDS Program

**DOI:** 10.4314/ahs.v21i1.7S

**Published:** 2021-05

**Authors:** Sara K Muli-Kinagwi, Meshack Ndirangu, Onesmus Gachuno, Samuel Muhula

**Affiliations:** 1 Amref Health Africa in Kenya; 2 College of Health Sciences, University of Nairobi

**Keywords:** Pediatric patients in care, Kibera community health center, HIV/AIDS

## Abstract

**Background:**

In 2011, 3.4 million children were living with HIV worldwide^1^.

**Objectives:**

Describe the characteristics of pediatric patients enrolled into the HIV program at the Kibera community health center between January 2012 and March 2013. Determine the proportion of enrolled paediatric patients lost to follow up. Determine the correlates associated with retention and loss to follow up

**Methods:**

The study was a retrospective cohort study of program data of all pediatric patients enrolled into the HIV program in the facility between January 2012 and March 2013. The data was analyzed using SPSS.

**Results:**

Of the 100 pediatric patients enrolled during the study period, 79 and 21 were HIV negative and positive respectively. Only 4 (5%) of the HIV exposed Infants and 11 (52%) of the HIV positive children were started on ART within the study period. The retention rate of the children at 3 months was 87% while the retention at both 12 and 15 months was 79%. There was an association between the mother or guardian disclosing their status and the retention of the child (p-value 0.026)

**Conclusion:**

The disclosure of the HIV status by parent/guardian to the child was associated with better retention of the children in the program.

## Introduction

Amref Health Africa in Kenya (AMREF) implements a unique care and treatment project in the informal settlement of Kibera in Nairobi with funding from the U.S. President's Emergency Plan for AIDS Relief (PEPFAR) through the Centers for Disease Control and Prevention (CDC) funding since 2003. In this project, AMREF provides support for HIV prevention, care, and treatment to adults and children, Tuberculosis (TB) diagnosis and treatment, and Prevention of Mother to Child Transmission of HIV (PMTCT) in four health facilities serving the Kibera slum. These services are provided using an integrated program comprising the comprehensive package for HIV/AIDS prevention, treatment and care among adults, children and infants^2^.

Kibera slum has a population of 170,000 people living in an area of approximately 2.5 square kilometers which is administratively organized into 13 villages^3^. The slum is one of the largest in Africa, with an estimated HIV prevalence of 12%. Social exclusion and economic deprivation in the midst of a relatively affluent city characterizes the Kibera slums. Not only is the population extremely poor (people live on USD 1.3 a day)^3^ but basic services such as water, sanitation, housing, and access roads are either lacking or of extremely low quality. The government has started infrastructural development initiatives that are slowly improving the face of the slum such as electricity, improved road network and several water and sanitation initiatives. However, providing adequate healthcare to its inhabitants still remains a major challenge.

In 2013, the project had cumulatively enrolled over 4,700 HIV patients on care and treatment since the project started with approximately 8% of the patients enrolled being children under the age of 15 years^2^. There are three categories of children enrolled into the HIV program: the HIV exposed child who has so far not yet had a HIV test or the previous test was negative but child is still breastfeeding; the HIV positive child who is on pre-Antiretroviral Therapy (ART) care and the HIV positive child who has started on ART.

The main cause of sub-optimal pediatric HIV care is the delay in diagnosis of HIV infection and the poor linkage of HIV positive infants to care and treatment. Many HIV infected parents who bring their children to Child Welfare Clinics (CWC) and general outpatient clinics do not know their own as well as their child's HIV status^4^. The World Health Organization (WHO) estimates that only 6–15% HIV exposed infants less than 1 year of age accessed EID programs in 2008–2010^5^. Children are thus fully dependent on their parents/guardians for their access to HIV care and treatment and the required follow up.

The pediatric age group includes children between the age of 0 years to 14 years. These may be children who were born to HIV infected women (vertically acquired HIV infection) or those who got infected during childhood or adolescence. Children born to HIV infected mothers need to be tested for HIV infection at 6 weeks of age through the DNA PCR test. For those who are DNA PCR negative at 6 weeks, repeat HIV rapid antibody tests at 9 and 18 months of age are carried out. A confirmatory PCR test is carried out for those who have a positive HIV antibody test at nine months of age. HIV infected children are required to be put on cotrimoxazole preventive therapy from 6 weeks of age.

The Kenya treatment guidelines stated that all HIV infected children below 2 years of age needed to be initiated on HAART immediately while for those above 2 years, treatment was dependent on their WHO stage and/or CD4 cell count^6^. The average retention rate of patients living with HIV/ AIDS on ART in the facility was at 86% in 2013 as reported by the project. However, the retention rate of HIV exposed children and those on care was not known by the project.

This study therefore looked at the retention rates of the pediatric patients enrolled in HIV care at the Kibera Community health center. For ART programs to succeed and improve the lives of HIV infected patients, it is important for the patient to be retained in the program and therefore benefit fully from treatment.

## Methods

The study was a retrospective cohort study of routinely collected program data of all pediatric patients enrolled into the HIV program in the Kibera community health center facility between January 2012 and March 2013. The study objectives were as below:

1. Describe the characteristics of pediatric patients enrolled into the HIV program at the Kibera community health center between January 2012 and March 2013
2. Determine the proportion of enrolled pediatric patients lost to follow up
3. Determine the correlates associated with retention and loss to follow up

Data abstraction was done from the facility's electronic medical record system as well as from the individual patient files for both the mother and the child. The collected data was edited manually before being analyzed using the SPSS-PC package. The data has been presented in total counts, percentages, charts and tables. The chi-square test of association was done to identify predictive factors to patient retention and loss to follow up. Some data relating to some of the variables of interest the study was looking at were not readily available from the records. The parent and child data were also not linked and hence was difficult to access additional information related to the parents of the enrolled children.

The Kibera project had ethical approval from the Kenya Medical Research Institute (KEMRI) ethics board and CDC to conduct studies using the clinical care database at the facility.

## Results

A total of 100 pediatric patients were enrolled between January 2012 and March 2013. Seventy-nine (79) children were identified to be HIV exposed children and twenty-one (21) were HIV positive children. Four (5%) of the HIV exposed children were started on antiretrovirals (ARVs) within the study period. Eleven (52%) of the HIV positive children were started on ART within the study period. The average time to starting ART was 2 months. The analysis thus focused on the remaining eighty-five (85) children who were either HIV exposed (75) and HIV positive (10) who were not on ART during the study period. Out of the 85 children, 51(60%) were male and 34(40%) were female.

**Figure 1:**
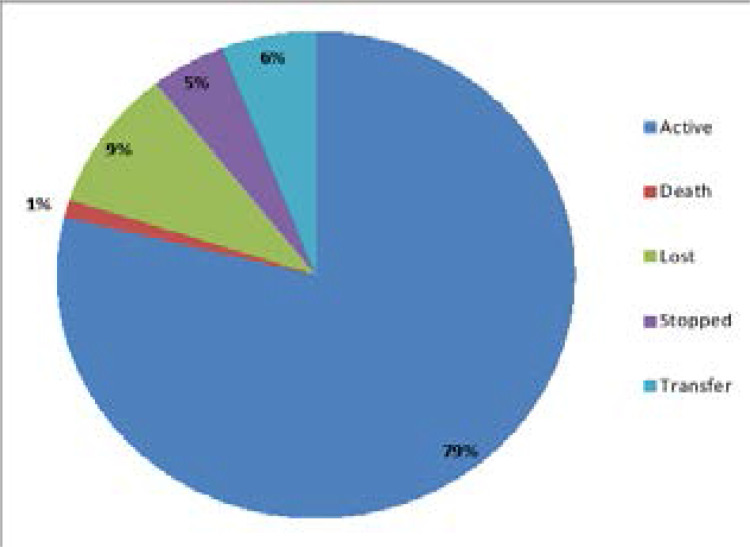
Child Treatment Status

Out of the 85 children, 67(79%) were active in the program while 8 (9%) were lost to follow up (LTFU), 5 (6%) had transferred out to another facility (TO), 4 (5%) had been stopped on ART while 1(1%) had died as shown in figure 1 below.

All the HIV exposed infants and HIV positive children were on cotrimoxazole. However, none of the 10 HIV positive children on pre-ART care had a baseline CD4 count done and documented in their file.

Nine (90%) of the HIV positive children on pre-ART care had been disclosed to their status but only one (1) child in this group was enrolled into a support group. There was no association between the mother/guardian's level of education or employment status and loss to follow up of the child.

There was no association between the facility where the mother/guardian was enrolled, the mother/guardian's level of education or the mother/guardian's employment status and the retention of the child in HIV care. There was an association between the mother/guardian disclosing her status and the retention of the child (p-value 0.026) as shown in table 2 below

**Table 2:** Tests of associations

Test of association at a confidence interval of 95%	P-value
Gender of the child and HIV Status of the child (Whether a child remains in the program or gets LTFU, dead or TO)	0.642
Age group of the child and Status of the child	0.135
Gender of the child and whether a child is LTFU or not	0.727
Age group of the child and whether a child is LTFU or not	0.213
Mother/guardian having disclosed status and nutritional status of the child	0.219
Mother/guardian enrolled in support group and nutritional status of the child	0.274
Mother/guardian level of education and nutritional status of the child	0.602
Mother/guardian employment status and nutritional status of the child	0.292
Child enrolled in support group and nutritional status	0.029
Mother/guardian disclosing status and LTFU	0.204
Mother/guardian enrolled in support group and LTFU	0.420
Mother/guardian level of education and LTFU	0.867
Child enrolled in support group and LTFU	0.586
Mother/guardian enrolled in support group and treatment status of the child	0.151
Mother/guardian level of education and status of the child	0.586
Child enrolled in support group and Status of the child	0.470
Mother/guardian disclosing status and retention of the child on Care	0.026
Mother/guardian getting enrolled in support group and retention of the child on care	0.564
Mother/guardian level of education and retention of the child on care	0.695
Mother/guardian employment status and retention of the child on care	0.171
Child nutritional status and retention of the child on care	0.01
Facility where the mother/guardian of the child was enrolled and retention of the child on care	0.682

## Discussion

The study showed that 8% of all patients ever enrolled on HIV care and treatment in the project were children under the age of 15 years. The study also showed that the baseline CD4 count of the 10 HIV positive children on pre-ART care had not been carried out and documented in their file. However, all the children who were on ART in the program had their CD4 counts available. This indicated that there was a delay in carrying out the CD4 baseline tests for these children. This was noted as a gap as it was important for the CD4 count of the HIV positive child to be done once the HIV diagnosis was made as part of the baseline preparation for ART. This would subsequently determine when ART would be started for those above 2 years of age at the time of the study.

The majority of the children enrolled over the period were retained in the program although the retention rate was noted to have dropped from 87% at 3 months to 79% at both 12 and 15 months. This raised concern as it was important to retain all the children in care so that they could be followed up and appropriate prevention interventions undertaken in a timely manner. In the event the child got infected with HIV, close follow up enabled the diagnosis to be made in good time and the child initiated on ART immediately thus preventing early morbidity and mortality.

The authors concluded that the project was losing on average 21% of its patients over a 15 months period with the main reason being due to loss to follow up. Due to the fact that the slum populations are highly ambulant, it was expected that transfer outs for care to other facilities were more likely. The loss to follow was therefore an area of concern for the project and a follow up study should be done to try and ascertain the factors that would lead to an increased number of lost to follow up among the pediatric patients.

Disclosure of HIV status by the mother or guardian was noted to be associated with retention of the child in the program. It was therefore crucial for the project to support parents and guardians to disclose their own HIV status to either family or friends and ensure they have been linked to a relevant support groups to enhance better retention of the enrolled children in the program. The HIV positive children also need to be disclosed to their HIV status at a suitable age and enrolled in support groups so that they may get the necessary psychosocial support as they continue to grow up and ensure that they are able to reach their full potential.

The study also found out that the treatment details of the parents and the children were not linked in the electronic medical records system for easier comparison and follow up of the parent-child pair. The HIV exposed infants (HEI) register had some details on the parents but further information which would be relevant to the child had to be accessed separately. It was therefore not possible to quickly get relevant details about the parents or guardians of a particular child which forced the research team to comb through the mother's files to access additional information.

The project needed to improve the data system by linking the file of the child with that of the parent electronically. This would enable the relevant details of both to be easily accessed and the two followed up as a par- entchild pair. This is important as the survival of the child is very dependent on the parent/guardian. In order to ensure that all the children enrolled in the program receive optimal care, it is important that all children once confirmed to be HIV positive should have a baseline CD4 test done and documented in their file.

## Recommendations

It is recommended that the project conducts further studies to identify the reasons for the loss to follow up of the enrolled children in the program. This will help the project design measures to address this and provide support to the parents as required so that the retention of the children is optimal and their quality of life enhanced.

## Figures and Tables

**Table 1:** Retention Rates in HIV Care

Time	Retention Rate
3 months	87%
6 months	87%
12 months	79%
15 months	79%
